# Analysis of Mechanically Activated Ion Channels at the Cell-Substrate Interface: Combining Pillar Arrays and Whole-Cell Patch-Clamp

**DOI:** 10.3389/fbioe.2019.00047

**Published:** 2019-03-22

**Authors:** Setareh Sianati, Anie Kurumlian, Evan Bailey, Kate Poole

**Affiliations:** ^1^EMBL Australia Node in Single Molecule Science, School of Medical Sciences, University of New South Wales, Sydney, NSW, Australia; ^2^Cellular and Systems Physiology, School of Medical Sciences, University of New South Wales, Sydney, NSW, Australia

**Keywords:** mechanically-activated ion channels, cell-substrate interface, electrophysiology, pillar arrays, protocol

## Abstract

Ionic currents can be evoked by mechanical inputs applied directly at the cell-substrate interface. These ionic currents are mediated by mechanically activated ion channels, where the open probability increases with increasing mechanical input. In order to study mechanically activated ion channels directly at the interface between cells and their environment, we have developed a technique to simultaneously monitor ion channel activity whilst stimuli are applied via displacement of cell-substrate contacts. This technique utilizes whole-cell patch-clamp electrophysiology and elastomeric pillar arrays, it is quantitative and appropriate for studying channels that respond to stimuli that are propagated to an adherent cell via the physical substrate. The mammalian channels PIEZO1, PIEZO2 have been shown to be activated by substrate deflections, using this technique. In addition, TRPV4 mediated currents can be evoked by substrate deflections, in contrast to alternate stimulation methods such as membrane stretch or cellular indentation. The deflections applied at cell-substrate points mimic the magnitude of physical stimuli that impact cells *in situ*.

## Introduction

Cellular mechanoelectrical transduction is the conversion of a mechanical stimulus into an electrochemical response. Such signal transduction is mediated by ion channels (pore forming proteins) that exhibit increasing open probability with increasing mechanical input to the cell (Martinac and Poole, 2018). Diverse functions have been attributed to signaling via mechanosensitive or mechanically activated (MA) ion channels in mammals. MA channel activity underpins our senses of touch (Delmas et al., 2011; Lechner and Lewin, 2013) and hearing (Fettiplace and Kim, 2014), is required for the development and homeostatic maintenance of the vasculature (Li et al., 2014) and recent data suggest that MA channels are activated by cell-generated forces (Pathak et al., 2014). In addition to their physiological function, MA channels with both gain-of-function and loss-of-function mutations have been shown to lead to pathophysiological disruption in numerous cells and tissues (Lamandé et al., 2011; Bae et al., 2013; Coste et al., 2013).

Not only are MA channels expressed in a diverse set of tissues, the mechanical environments and stimuli applied to cells are also highly varied. For instance, the chondrocytes in articular cartilage are impacted by compressive forces during joint movement, combined with shear forces as fluid moves through the joint. In addition, there are tensile forces propagated to the cells via the extracellular matrix within which the individual cells are embedded (Guilak et al., 1995; Sanchez-Adams and Athanasiou, 2011; Madden et al., 2013). At the cellular level, these distinct forces will lead to cellular compression, stretch of the cell membrane and pulling at regions of contact between cells and their surrounding matrix (referred to here as the cell-substrate interface). Similarly, during tumor development and metastasis cancerous cells experience varied mechanical environments. The mechanics at the primary tumor site reflect changes in matrix production, leading to a reduction in the compliance of the tumor microenvironment. Any invasive cells that metastasize away from the primary tumor must navigate complex and diverse environments that often require cells to enter a state of confinement (Paul et al., 2017; Van Helvert et al., 2018). The mechanosensitivity of non-motile cells can also be impacted by the underlying substrate. For instance, in sensory neurons the specific laminin substrate can locally polarize neuritic segments into active and inactive regions (over EHS-Laminin and Laminin-332, respectively) (Chiang et al., 2011).

Given the diversity of mechanics and forces that cells experience it is important to study whether MA channels are ubiquitously activated by a spectrum of mechanical inputs or whether they respond to a restricted set of mechanical cues. This then presents a challenge of how to study MA channel activity within the appropriate context. Ion channel activation leads to the passive diffusion of selected ions down their electrochemical gradient (Moorhouse, 2015), through the pore of the channel (most mammalian ion channels have a selectivity filter that limits the specific ions that can traverse the pore). In order to study channel activity, regardless of ionic permeability, the gold standard technique is to use patch-clamp electrophysiology. This set of related techniques enables the direct measurement of ionic flux across the membrane. However, in order to study mechanically activated ion channels, patch clamp measurements need to be combined with the application of a mechanical stimulus to the patched cell.

For many years the application of stretch to the membrane via the patch pipette itself has been used to study MA channels. The first measurement of mechanical activation of single channels in the cell membrane was conducted by Guharay and Sachs, by applying an increase in suction to membrane patches from chick skeletal muscle cells (Guharay and Sachs, 1984). Today, high-speed pressure-clamp (HSPC) (Besch et al., 2002) is used as a routine analysis to study such mammalian MA channels as PIEZO1 and TREK-1 and TRAAK (Coste et al., 2010; Brohawn et al., 2014; Moroni et al., 2018). As a complementary method to study macro currents, whole-cell patch-clamp is combined with the indentation of the cell with a glass rod (Drew et al., 2002; Hu and Lewin, 2006). The mammalian channels PIEZO1 and PIEZO2 will respond to such stimuli (Coste et al., 2010; Dubin et al., 2017). The MA channels identified to date cannot account for the diversity of currents evoked using cellular indentation, suggesting that there are additional MA channels that have not yet been identified.

Whilst both HSPC and cellular indentation have generated much interesting data and significantly advanced our understanding of the expression pattern and activity of MA channels, both approaches apply stimuli to the apical surface of the cell. As such, neither technique directly addresses MA channel activation at the interface between cells and their substrates. As mentioned above, this cellular compartment is critically important in cellular mechanosensing in a number of cells and tissues. In order to directly apply stimuli at this interface we developed a technique whereby mechanical stimuli can be applied to cells cultured on elastomeric pillar arrays (Poole et al., 2014). This technique represents a modification of the approach previously developed to quantify cell-generated forces (Tan et al., 2003; du Roure et al., 2005; Ganz et al., 2006; Desai et al., 2007). The channel activity in cells cultured on the arrays is monitored using whole-cell patch-clamp and stimuli are applied by deflecting an individual pilus subjacent to the cell. Pillar deflection has been shown to evoke PIEZO1, PIEZO2 (Poole et al., 2014) and TRPV4 (Servin-Vences et al., 2017; Tay et al., 2018) mediated currents and to evoke MA channel activity in primary cells, such as somatosensory neurons and chondrocytes (Poole et al., 2014; Servin-Vences et al., 2017, 2018; Wetzel et al., 2017). The technique is quantitative and preserves transmembrane force-sensing structures incorporating the substrate, extracellular matrix (ECM), cell attachments and intracellular components, such as STOML3, that can tune the sensitivity of the MA channels (Poole et al., 2014; Wetzel et al., 2017). As such, this technique can directly probe MA channels within the appropriate cellular context and be used to study how regulatory proteins modulate channel activity in intact force-sensing complexes.

### Experimental Design

Cells are cultured on pillar arrays of defined dimensions. Once adhered, a high-resistance giga-Ohm (GΩ) seal is formed between a patch-pipette and the cell, the region of membrane within the patch pipette is then disrupted to enable direct fluid access between the solution in the pipette and the intracellular space. In this whole-cell patch-clamp mode any channel activity that leads to a net flux of ions across the plasma membrane can be measured. To apply stimuli, a glass probe driven by a precisely controlled manipulator is positioned adjacent a pilus located subjacent to the patched cell. A series of deflection stimuli is then applied to the cell by deflecting the pilus across the range of 1–1,000 nm. Images are acquired during the stimulations from which precise stimulus sizes can be calculated in a *post-hoc* analysis. Using this approach MA channels can be activated with molecular-scale inputs, that are applied directly at the interface between cells and their substrate.

### Advantages and Limitations of Approach

The main limitation of this experimental approach to studying MA channel activity is that it can only be utilized to study channel activation in adherent, dissociated cells that express MA channels at sufficiently high levels to allow detection of macroscopic currents. As such, *ex vivo* and *in vivo* recordings are not supported. In addition, whilst defined, quantifiable stimuli can be applied to cells, it is not possible to derive how much force impacts the MA channels themselves. This limitation is shared with the other well-established methods for evoking MA currents: In the case of cellular indentation, the contact area between stimulator and cell is unknown, the curvature of the indented membrane and the point at which the stimulator contacts the cell; in the case of HSPC, elegant experiments have been used to estimate the membrane tension required to activate PIEZO1 in membrane blebs (Cox et al., 2016), however this simplified system does not reflect the native environment of PIEZO1 *in situ*. The advantages of our approach lie in the fact that stimuli are applied via connections between cells and their substrates. The design of the experiment enables a dissection of the diverse factors that can regulate MA channel force sensing: that is, the mechanics of the substrate can be modulated, the pillar arrays can be coated with distinct ECM molecules and cellular components can be manipulated using standard molecular biology techniques. The preservation of these transmembrane structures means that the MA channel activity can be studied in an appropriate mechanical context. In addition, we have found that TRPV4-mediated currents are not robustly evoked by HSPC and not evoked at all by cellular indentation, yet pillar deflection evoked sensitive TRPV4 mediated currents. As such, there are MA channels that are only activated when stimuli are applied directly at the cell-substrate interface.

## Materials and Equipment

### Materials

- Positive, microfabricated masters to cast pillar arrays. These masters can be ordered from companies such as Bonda Technology Pte Ltd. (Singapore). Specifications of the masters that we have used are presented in Table S1. Key attributes are that pili should be at least 5 μm high.
- Glass coverglass, 22 × 22 mm, Thickness 2 (VWR, 631-0126)
- Glass coverglass 13 mm diameter, Thickness 1.5 (Menzel Glaeser, ThermoFisher Scientific, MENCSC1315GP)
- Fast curing, 2-component epoxy (Selley's 5 min Araldite, 9300697106391)
- Plastic petri dishes, 35 mm × 10 mm (Corning, 430165)
- Thick walled, filamented capilliary glass (SDR Scientific, 30-0060, GC150F)
- Microfil needles, 28 gauge/ 97 mm long (World Precision Instruments, MF 28 G - 5)

### Reagents

- Cells: Any adherent cells can be tested using this protocol. Cells should be maintained in appropriate media. Proliferative cells should always be passaged before reaching 100% confluence and primary cells should be carefully isolated so as not to damage the cell membrane.
- Polydimethyl Siloxane (PDMS, Sylgard 184)
- Trichloro(1H,1H,2H,2H-perfluorooctyl)silane, 97% (Sigma-Aldrich, 448931). CAUTION: in both liquid and vapor phase this silane is toxic and corrosive.
- Cell dissociation solution, Non-enzymatic (Sigma-Aldrich, C5914)
- NaCl (Ajax Finechem, AJA465)
- KCl (Chem-supply, PA054)
- CaCl_2_ (VWR Chemicals, 22317.260)
- MgCl_2_ (Ajax Finechem, AJA296)
- D-Glucose, anhydrous (Chem-supply, GA018)
- HEPES ≥99.5% (Sigma Aldrich, H3375)
- EGTA > 97% (Sigma Aldrich, E4378)

#### Optional Reagents

- Recombinant human laminin (specific isoform will depend on experiment) (BioLamina, Sweden)
- Fibronectin, pure (Sigma Aldrich, 11051407001)
- Poly-L-Lysine, 0.01% (Sigma-Aldrich, P4707)
- Lucifer yellow, 3% in intracellular buffer (Sigma-Aldrich, L0259)
- Fugene HD (Promega, E2311)
- Lifeact-eGFP (e.g., #54610, Addgene), Lifeact-mCherry (e.g., #54491, Addgene) encoding plasmids

### Reagent Preparation

- The intracellular buffer (IC) is prepared using ultrapure water with the following components: NaCl (10 mM), KCl (135 mM), MgCl_2_ (1 mM), HEPES (10 mM), EGTA (1 mM).
- The extracellular buffer (EC) is prepared using ultrapure water with the following composition: NaCl (140 mM), KCl (4 mM), CaCl_2_ (2 mM), MgCl_2_ (1 mM), Glucose (4 mM), HEPES (10 mM)
- For both IC and EC buffers it is essential to adjust the pH and the osmolarity. For the EC solution the pH should be adjusted to 7.4 using sodium hydroxide. For the IC solution the pH should be adjusted to 7.2–7.3 using potassium hydroxide. To prepare the buffers as accurately as possible, first dissolve the reagents in 70% of the final volume of milli Q water. Once the desired pH is achieved, adjust the buffer volume in a volumetric flask. It is important to keep the pH consistent throughout the experiments, as it may affect the channel functions. Finally, adjust the osmolarity of the solutions to protect the plasma membrane from the excessive osmotic forces. The IC solution osmolarity should be 10–20 mOsm lower than for the EC solution. This difference increases the rate of success in forming a GΩ seal. The above recipe should result in an observed osmolarity of around 280 and 290 mOsm for IC and EC solutions, respectively. These values differ from the osmolarity predicted for ideal conditions (304 mOsm and 311 mOsm, respectively), due to the osmotic coefficients of the individual solutes. Osmolarity must be controlled with osmometer every time the buffers are made. Sucrose can be used to increase the osmolarity while keeping the ion concentrations constant. It is preferable to filter the solutions by passing through a 22 μm filter. IC solution can be dispensed at 1 mL aliquots and stored at −20°C until use. The EC buffer can be stored at 4°C up to 2 weeks.

### Equipment

- Vacuum dessicator
- Oven
- pH meter
- Osmometer
- Low pressure plasma system (Diener electronic, Zepto ONE)
- Pipette puller (Sutter, P-1000)
- Microforge
- Whole-cell patch-clamp equipment installed on an inverted light microscope (Nikon, *Ti*U), fixed to an optical table to dampen vibrations and enclosed in a Faraday cage to minimize electrical noise. A micromanipulator is required to control the movement of the patch-clamp headstage (Scientifica, PatchStar). A long-distance 40x objective with an adjustable coat and a camera [CCD or sCMOS, (Nikon DS-Qi2 CMOS)] with pixels smaller than 8.5 × 8.5 μm are required for acquiring images. Electrophysiological recordings are obtained using an amplifier, with appropriate software and a digitizer [Axopatch 900B controlled by pClamp10 software and a Digidata 1550B digitizer (Molecular Devices)]. Electrodes made from chlorinated silver wire (99.99% purity).
- Nano-stimulator (Kleindiek, Germany, MM3A-LS).

## Stepwise Procedures

### Design and Order Positive Masters

Positive microfabricated masters should be designed as these silicon masters can be fragile: creating positive microfabricated masters minimizes handling. In order to culture cells and apply deflection stimuli there are some constraints on the dimensions of the elements of the array. We routinely use arrays with individual pili that are 5 μm high. The diameter and center-to-center spacing of individual pili can be varied, see Table S1 for validated dimensions. Increasing the length and/or decreasing the diameter of pili can lead to arrays that collapse when removed from master and should be avoided.

Pause point: Once positive masters have been obtained they can be reused and will only need to be replaced if damaged or if a new design is required.

### Prepare Negative Masters

**Figure 1:**
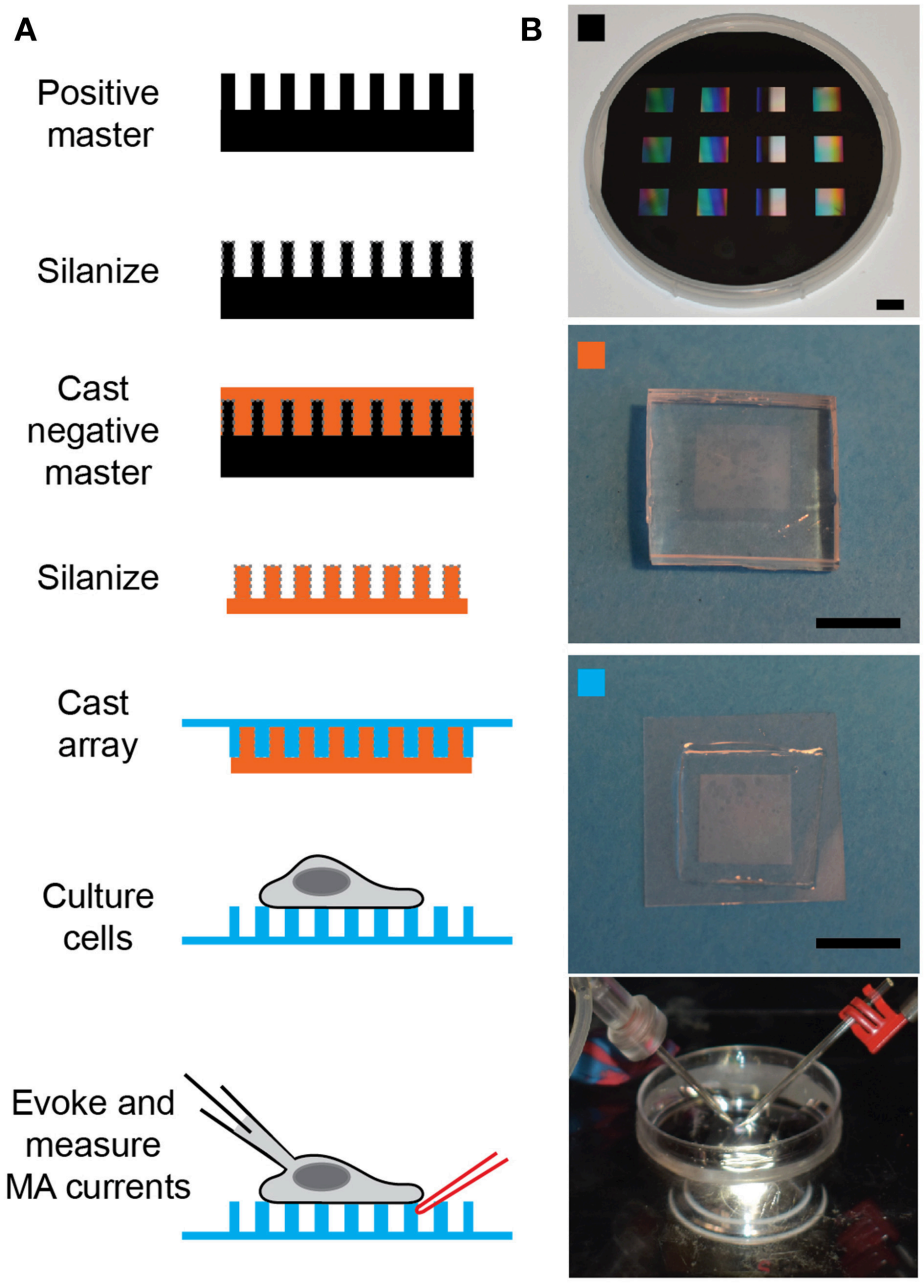
Preparing pillar arrays**. (A)** Flow diagram outlining the steps from obtaining the positive master to analyzing MA channel activity in cells. The positive master is silanized before casting a negative master in PDMS. These negative masters are then silanized and used multiple times to cast arrays in PDMS, with a glass backing. Cells can be cultured on these arrays and then analyzed by deflecting a pilus subject to the cell with a glass probe (in red) whilst the cell is simultaneously monitored using whole-cell patch-clamp (pipette in black). **(B)** The top panel is a photograph of a positive master, then marked with an orange square is a photograph of a negative master. The panel marked with a cyan square shows a pillar array and the bottom channel shows the dish mounted on the microscope with the patch pipette on the left and the stimulating pipette mounted in the stimulator on the right. Scale bars = 10 mm.

In order to create negative masters from which to cast positive arrays, the positive master is first silanized (Figure 1). Carefully place positive master, structured side up, in a vacuum dessicator, in a fume hood. Place clean glass coverslips to either side of the master and place a 5 μl of drop of Trichloro (1H,1H,2H,2H-perfluorooctyl) silane on each of the two coverslips. Close the dessicator, apply vacuum and leave in fume hood overnight.

CRITICAL STEP: it is essential to ensure that the microfabricated master is properly silanized each time before casting negative masters. If the silanization does not create a sufficiently hydrophobic surface the negative masters will not easily peel away and the positive master may end up damaged.
CAUTION: the positive masters are fragile and should be handled with care.
CAUTION: Trichloro(1H,1H,2H,2H-perfluorooctyl)silane is toxic and corrosive in its vapor and liquid phases. Always handle in a fume hood and carefully flush the bottle with an inert gas, such as nitrogen, before storing.

After overnight silanization, remove the glass coverslips on which the Trichloro(1H,1H,2H,2H-perfluorooctyl)silane was placed and dispose of appropriately. Please note that the silane deposited on the surface of the master is no longer toxic. Carefully transfer the positive master to a disposable, flexible, heat-resistant vessel (we use 3 stacked, large weigh-boats with a high heat tolerance). Test the silanization of the master by placing a 10 μl drop of milliQ water on the surface. If the contact angle is high the silanization is effective, if the contact angle is low repeat the silanization step.

Prepare 30 mL PDMS from the two-components provided in a 1:10 ratio of curing agent:elastomer, mix extremely well and degas for 30 min. While PDMS mixture is degassing turn oven on to 80°C. When PDMS is degassed gently pour still fluid mixture over the silanized master to a depth of 4–5 mm. Place into oven and cure for 15 min.

After curing remove from oven and very gently cut the weigh boats away from the master and PDMS. Allow to cool and turn the sandwich over. Trim excess PDMS away from the underside of the master and then turn back over so that the master is on the underside of the PDMS. Gently deform the PDMS at one edge, this should cause the PDMS to easily pull away from the master. Keep gently deforming the PDMS until the master has been completely released from the PDMS. Return master to secure, dust-free storage container until next required.

CAUTION: The PDMS should only be 4–5 mm thick and should be very gently deformed so as not to destroy the master. Take care to ensure that that when the positive master comes away from the PDMS it does not fall as it will shatter. It is recommended to have the PDMS-master sandwich sitting on a clean bench and then gently deform the PDMS up to release the master so that the master stays flat against the bench.

Immediately return the positive master to a secure, clean storage space.

Take block of PDMS and excise individual negative masters using straight-edge razor blade.

Pause point: these negative masters can be stored indefinitely in a clean environment for future use

Silanize overnight using Trichloro(1H,1H,2H,2H-perfluorooctyl)silane, as above. Note: these negative masters will be used to cast the experimental arrays, they can be used for multiple casts, but will need to periodically be re-silanized. The frequency of silanization depends on climate, in dry locations silanization every 4–5 casts is sufficient, in more humid environs silanization may need to be repeated after every use. Examples: Berlin, Germany every 5 casts, Sydney, Australia every 3 casts, Singapore, every cast.

### Cast Pillar Arrays

Mix PDMS at a ratio of 1:10, curing agent:elastomer and mix thoroughly before degassing for 30 min. Whilst PDMS is degassing turn oven on to 110°C. Place silanized, negative PDMS masters patterned-side up onto a glass dish or tray. After degassing carefully drop fluid PDMS onto the top of the master (try to avoid touching the structure so that masters last longer). Spread PDMS over the top of the master and leave to sit for 30 min.

Note: 4 mL PDMS is more than sufficient to cast 12 arrays.

Activate a coverslip (22 × 22 mm, Thickness 2) for each array by placing in a plasma system and treating with oxygen plasma for 90 s. Immediately after activation place the coverslip over the PDMS-coated master and gently apply pressure so that there is a thin layer of PDMS between coverslip and master. Place the master-PDMS-coverglass sandwich in the oven for exactly 1 h.

Note: a similar elasticity (2.1 MPa) of the PDMS can be achieved by curing for 16 h at 60°C (instead of 1 h at 110°C).

After curing, remove the master-PDMS-coverglass sandwich from the oven and very carefully peel the master away from the coverslip. If the master is sufficiently silanized the PDMS should readily peel away. If, however, the silanization is insufficient, it will be more challenging to remove the PDMS and increase the likelihood of damaging the master.

Pause point: Store freshly cast pillar arrays in a clean, covered environment, such as a large petri dish and use within 1–2 weeks of casting.

### Preparing Arrays for Cell Plating

Use two-component epoxy to affix array in the bottom of a dish of appropriate size to mount on the microscope fitted with patch clamp and stimulator (we use 35 mm petri dishes from Corning). Use four small dots of epoxy at the corners of the underside of the glass on which the pillar array has been cast and endeavor to apply even amounts of epoxy at each corner. Affix array in petri dish and allow to cure before moving on to next step (5 min for fast curing epoxy).

There are a number of ways to prepare the arrays for cell culture- each will depend on the cell type to be studied. We provide here options for uncoated, globally coated and coated at the tops of the arrays.

#### Uncoated Arrays

Adherent cells will attach to uncoated PDMS, particularly if the PDMS has been activated. Place array in plasma system and treat with oxygen plasma for 90 s. Within 30 min of this treatment, plate dissociated cells directly onto activated PDMS.

Note: this approach has been successfully used to study MA currents in chondrocytes, HEK-293T cells heterologously expressing MA channels and some cancer cell lines.

#### Globally-Coated Arrays

In order to investigate the role of specific ECM molecules in regulating MA channel activity, the pillar arrays can be globally coated.

Prepare a solution containing the protein of interest: laminin isoforms at a concentration of 10 μg/mL, fibronectin at 10 μg/mL or poly-L-lysine at 0.01% in PBS. Activate the arrays using oxygen plasma for 90 s and then place a drop of the protein solution on the array. Incubate for 1 h in a humidified incubator. Gently wash the array with media before plating dissociated cells on top of the array.

#### Coating Exclusively the Tops of the Arrays

In some cases, it is best to restrict the ECM coating exclusively to the top of the pillar structures. This approach is important when studying neuronal cells where neurite outgrowth needs to be restricted just to the tops of the array.

Option 1*:* Treat arrays with oxygen plasma and then leave in a sterile environment for 1 h to allow the surface to repassivate. Silanize the arrays with Trichloro(1H,1H,2H,2H-perfluorooctyl)silane for exactly 30 min. This treatment will render the array hydrophobic. Place a drop of solution containing ECM protein (see above) on the top of the array and due to the hydrophobicity the droplet will sit on top and not flow between the structured elements. Carefully cover the droplet with a small, round glass coverslip (13 mm diameter) and leave overnight in a humidified incubator. Remove the small coverslip in the morning and then wash the array with media.

Note: it is best to leave the array submerged in cell culture media for 12–24 h to reduce the hydrophobicity of the array before plating cells.

Note: care must be taken exchanging media and buffers on these arrays as it is easy to strip all the cells off the surface if the hydrophobicity drives the liquid away from the structured area.

Option 2*:* Prepare some blocks of PDMS that are slightly larger than the structured area of the array. In this case, prepare the PDMS mixture at a ratio of 1:20 curing agent:elastomer. After degassing, cure at 110°C for 15 min. The PDMS will remain a little sticky when removed from the oven. Cut the PDMS into blocks slightly larger than the array.

Coat the PDMS blocks with the solution containing the ECM molecules (see above) and incubate for 30–60 min in a humidified incubator. Collect the excess ECM solution from the blocks (this remainder can be stored for 1 week and reused), rinse PDMS blocks with ultrapure water and dry under a stream of nitrogen. Activate the pillar array using oxygen plasma and then immediately apply the PDMS cubit, ECM coated side down, to the tops of the array. Gently apply pressure to gain a good contact between PDMS and pillar array, without disrupting the array itself. Leave for 30 min in humidified incubator before removing the PDMS cubit. These arrays are now ready for cell culture.

Note: we have found that option 1 gives a more even coating of ECM molecules [as have other researchers (Ganz et al., 2006)] but arrays prepared in this fashion are more difficult to handle, due to the increased hydrophobicity.

### Culturing Cells on Arrays

Adherent cells can be studied with this technique, preparation of cells for plating on arrays will depend on timing and cell type.

For freshly isolated primary cells (Servin-Vences et al., 2017; Wetzel et al., 2017): isolate cells with standard protocols but avoid mechanical damage or disruption of membrane integrity so as to avoid disrupting the formation of a tight seal during patch-clamp analysis.

For cultured cells: If experiments are to be conducted acutely (within hours of preparation) release cells from tissue culture plastic using non-enzymatic cell dissociation solution, if experiments are to be conducted the following day, standard trypsin-based protocols can be used. A critical consideration when working with cultured cells is to ensure that they never grow past confluence, for most cultured cell lines this will reduce the ease of forming a high-resistance seal between the patch pipette and the cell membrane.

It is recommended to transfect cells with a plasmid encoding Lifeact-eGFP or Lifeact-mCherry to be able to accurately visualize the boundaries of the cell to avoid hitting the cell or any fine filopodia that may extend from the cell body. For terminally-differentiated cells that are more challenging to transfect a membrane impermeable dye, such as lucifer yellow, can be included in the patch pipette, such that the intracellular space of a cell in whole-cell mode is rendered fluorescent.

Cells should be studied within 36 h of plating to optimize patching conditions. Primary cells may need to be analyzed on the day of deposition, depending on propensity to de-differentiate (Servin-Vences et al., 2017).

### Whole-Cell Patch-Clamp

Prepare glass pipettes for patching. The glass used for and shape of the patch pipettes are critical variables. We use the Sutter P-1000 puller fitted with a 2.5 mm box filament (SDR Scientific, FB255B) and thick-walled, filamentous glass (SDR Scientific, 30-0060, GC150F) to create pipettes with a resistance of 3–6 MΩ. These pipettes are fire polished with a home-made microforge before use.

Glass pipettes can be prepared the day prior to patching and stored in a dust-free container. Care should be taken to not touch the fine tip of the pipette against any surface as they are fragile and will break easily. It is critical that the end of the pipette is free from structural defects.

Prepare glass probe for pillar deflections. We convert the same types of pipettes created for patching into stimulators by heating the end with the microforge to seal the tip. Care should be taken to make sure the end is sealed, otherwise the edges of the glass can damage the cell. The tip should be no larger than 1–2 mm, if it gets too broad it is difficult to deflect pili without disrupting the cell. Glass stimulating probes can be prepared in advance and stored in a dust-free environment indefinitely until use, they can be reused until they are damaged.

Prepare both intracellular and extracellular solutions and bring to room temperature. (Pre-prepared IC buffer can be filtered, aliquoted and stored for 12 months at −20°C, EC buffer can be prepared in bulk, filtered and stored at 4°C for 1–2 weeks.)

Gently wash all media off the cells, add extracellular buffer to the dish and mount the dish on the inverted light microscope. Insert the reference electrode into the dish, making sure that it is submerged under the level of the buffer.

Change to a low power objective (10x) and select a cell that connects to pili that can be accessed with the stimulator without hitting another region of the cell. It is important to select individual cells.

Mount a glass stimulating probe in the MM3A-LS and position it so that the shadow of the glass is visible in the field of view before carefully maneuvering the stimulator close to the cell that will be patched. It is not recommended to position the stimulator directly at the pilus before patching the cell but it should be visible within the field of view to enable final positioning after the whole-cell patch configuration has been established for the selected cell.

Fill the end of a glass pipette with intracellular buffer using a syringe fitted with a very fine, long needle (only add buffer to fill the end of the pipette, as increased buffer will increase noise). Check that there are no bubbles in the pipette and mount into the pipette holder of the headstage, taking care to tighten the holding screw to ensure that the pipette is stable and sealed. Swing the manipulator over the dish and before lowering it into the extracellular buffer apply a small amount of outward pressure.

Lower the patch pipette into the extracellular buffer and note the resistance of the pipette when a test pulse is applied at a holding potential of 0 mV. If the resistance is outside 3–6 MΩ, release the positive pressure, retract and discard the pipette before starting again.

CAUTION: if the pipette does not have the desired resistance ensure that the pressure is released before trying to remove the pipette, otherwise the pipette may be expelled from the headstage holder due to the outward pressure.

Once a pipette of appropriate resistance is mounted in the headstage holder, use the course movement to position the pipette over the cell to be patched. The pipette should be oriented such that contact will be made close to the apex of the cell.

**Figure 2:**
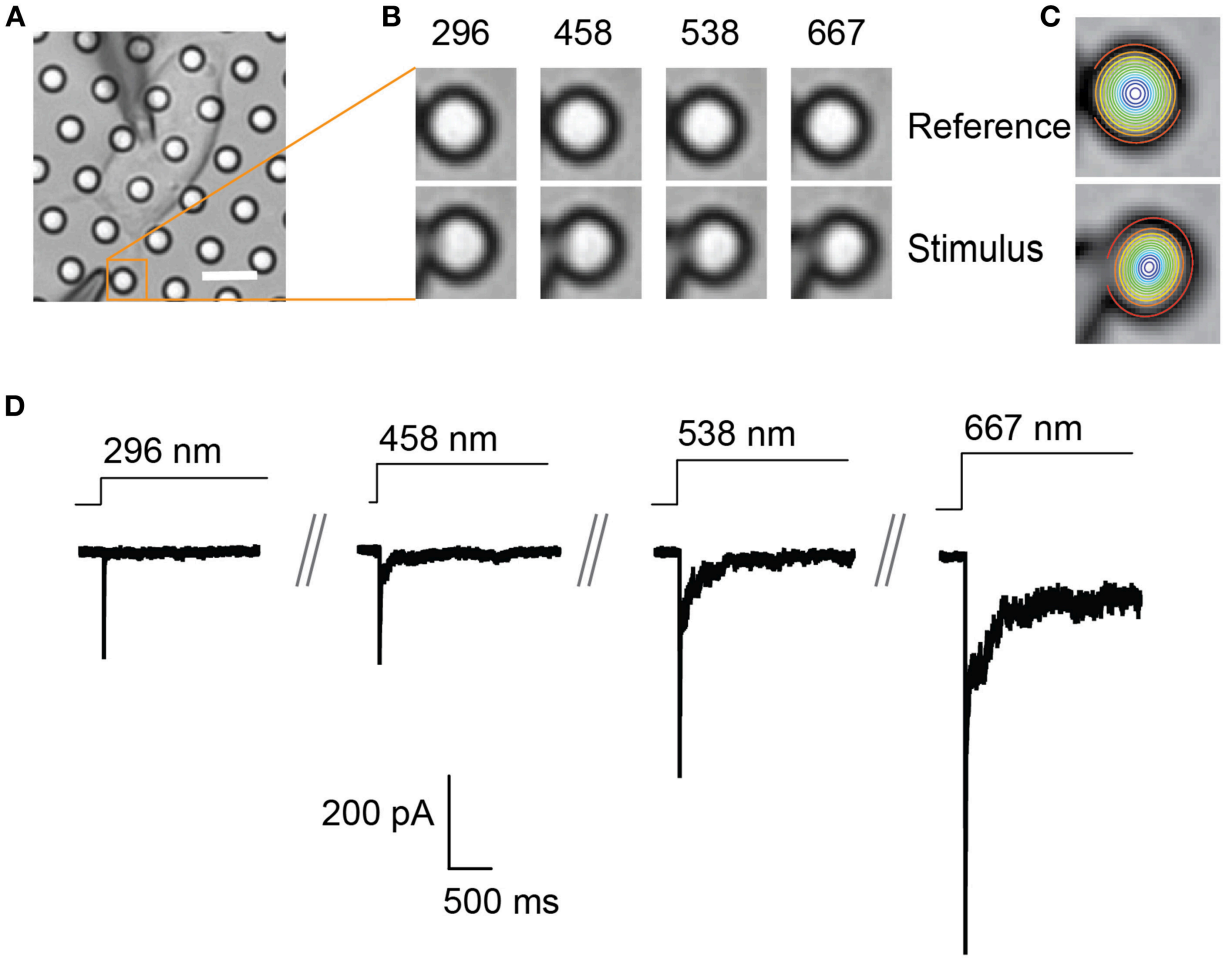
Representative pillar deflections and corresponding TRPV4-mediated currents**. (A)** Bright-field image of a single HEK-293T cell expressing TRPV4 cultured on the pillar array, scale bar 10 μm. **(B)** Series of mechanical stimuli are applied directly at cell-substrate interface by deflecting the pilus subjacent to the cell (orange box). Middle panel shows the movement of indicated pilus in response to the stimuli of increasing magnitude from 296 to 667 nm. **(C)** The center point of the pilus is determined from a 2D Gaussian fit of intensity values in the images of before and during deflection. **(D)** Representative traces of TRPV4-mediated currents corresponding to the stimuli presented in **(B)**.

Move the 40x objective into place and ensure that the microscope is set for bright-field contrast. At this point it is essential to ensure that the camera will capture well-contrasted bright field images with the focus set to the top of the pili (Figure 2A). Poor bright field images will hamper the analysis of pillar movement and any adjustments of the microscope at a later stage risk disrupting the high-resistance seal between the membrane and the pipette.

Ensure that the amplifier is in “patch” mode. Approach the cell using the fine movement of the manipulator controlling the movement of the headstage until there is a 10% increase in the pipette resistance, release the outward pressure and apply inward pressure in order to form a high resistance seal between the pipette and the membrane (>1 GΩ). Use the Fast and Slow compensation to charge the pipette capacitance, adjust the holding potential to −60 mV [or appropriate voltage is the cells to be studied exhibit a different transmembrane potential, i.e., chondrocytes −40 mV (Servin-Vences et al., 2017)] and use a quick pulse of inward pressure to rupture the membrane patch. Switch the amplifier to “whole-cell” mode and use the whole-cell compensation to charge the membrane capacitance. Once the whole cell compensation has been adjusted appropriately, the series resistance should be compensated to at least 60%. For further details on the intricacies of whole-cell patch-clamp, the Axon Guide (available from the Molecular Devices website: www.moleculardevices.com) provides detailed discussion.

### Applying Stimuli at the Cell-Substrate Interface

Once the cell is in whole-cell patch-clamp mode, the stimulator should be already near the cell. Start recording in voltage-clamp mode (at appropriate holding potential for the cells being studied, e.g., sensory neurons −60 mV, chondrocytes −40 mV). Finish positioning the stimulator adjacent a pilus that lies subjacent to the cell, while monitoring the voltage clamp recording to control for whether the stimulator hits the cell during the final positioning. Ensure that the stimulator will not hit any part of the cell or any fine filopodia that may extend from the cell body (cells expressing a fluorescent marker such as Lifeact-eGFP or Lifeact-mCherry will enable clear visualization of the cell boundaries).

#### Collecting Data

Start by acquiring an image of the cell before stimulation. Begin a voltage-clamp recording and then initiate a series of deflection stimuli (Figure 2B). Each stimulus should be applied for a minimum of 0.5–1 s to enable acquisition of an image during each individual stimulus. A pause of 10 s between each stimulus should be employed to avoid current rundown over the course of the experiment (for each MA channel/cell type this delay should be empirically determined). It is recommended to also acquire an image between each stimulus to act as a reference for the following stimulus, thus accounting for any drift of the dish during the experiment.

In order to calculate a threshold of channel activation, apply a series of stimuli of increasing magnitude, from a few nm up to 1,000 nm. In order to generate stimulus-response curves, randomize the stimuli. It is important to apply at least 5 stimuli at a pilus that span the stimulus range. In addition, there is pilus-to-pilus and cell-to-cell variation as the stimulus region contains a restricted number of channels. As such, it is best practice to apply a series of stimuli to at least 2 pili subjacent to each individual cell. In particular, when no response is noted at an individual pilus a second pilus should be sampled.

### Analyzing Data

**Figure 3:**
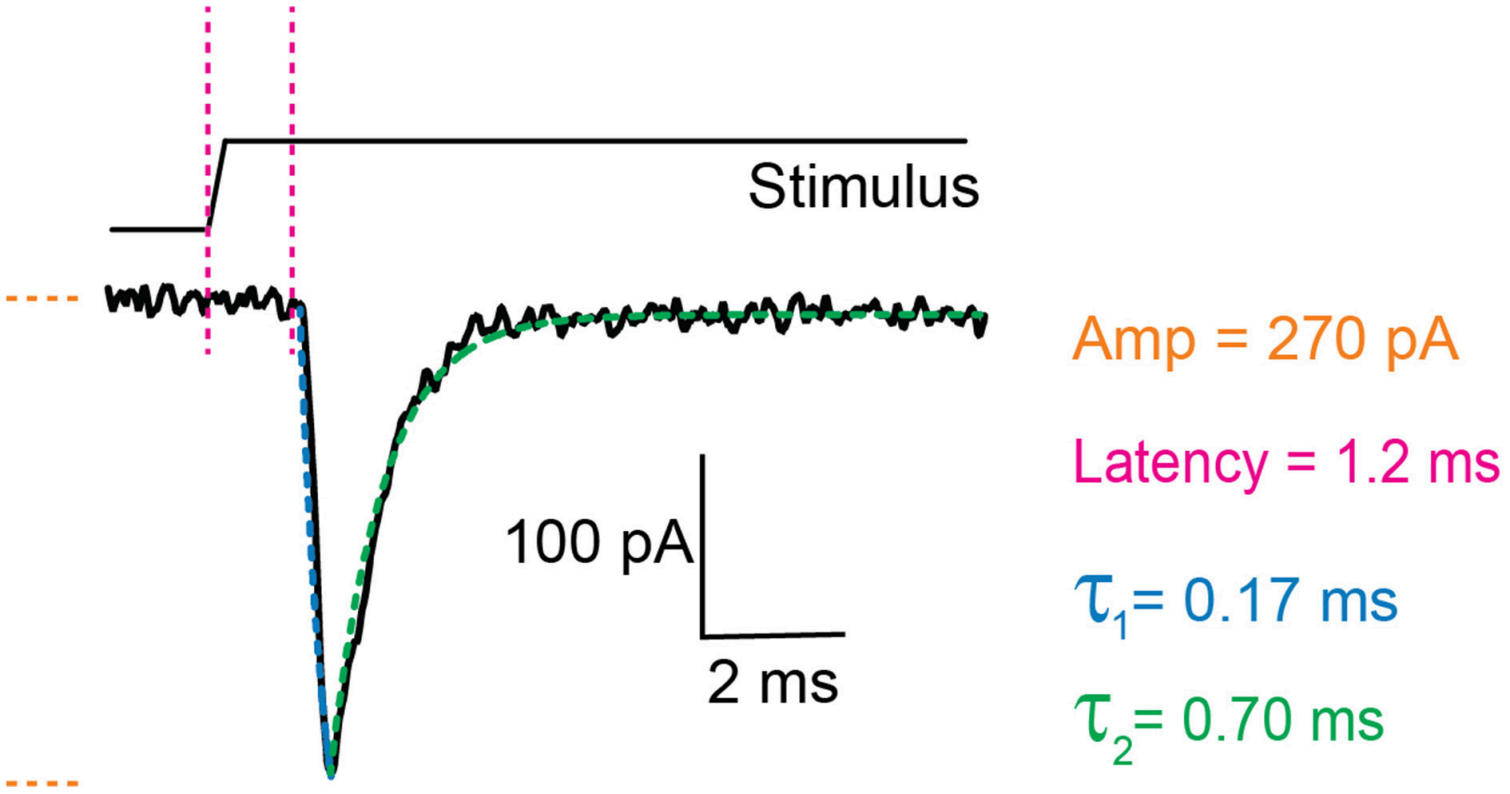
Example trace of a mechanically activated current. Example trace of a deflection-gated response. Marked in orange is the amplitude of the current, in magenta the latency between stimulus and onset of the response, in blue the activation time constant (τ_1_) and in green the inactivation time constant (τ_2_). These data were generated in HEK-293T cells expressing TRPV4.

There are two components to the analysis, characterizing the current parameters and quantifying stimulus sizes. The voltage-clamp recordings are opened in the Clampfit software and the following parameters are analyzed: current amplitude, latency, activation time constant (τ_1_) and inactivation time constant (τ_2_) (Figure 3). Current amplitudes are measured from the pre-stimulus baseline amplitude to the peak of the current. The latency is measured from the start of the stimulus to the onset of the current. The activation and inactivation time constants are calculated from a mono-exponential fit of the current rise time and current decay.

In order to quantitate the stimulus magnitude (pillar deflection) it is necessary to determine the center point of the deflected pilus from the images acquired during the experiment. Each individual pilus acts as a light guide. As such, the relative x-y co-ordinates that correspond to the center of the pilus can be calculated from a 2D Gaussian fit of intensity values. This can be achieved using the in-built analysis routines of the Igor Pro 7 analysis software (WaveMetrics Inc.). The center point of the pilus in successive images, taken before, during and after the stimulus, can then be used to calculate the exact deflection applied (Figure 2).

## Timing

### Pre-experiment Preparation

Three weeks: design of pillar array masters and manufacture. Note, the masters generated in this step can be reused multiple times if handled with care. This step should not need to be repeated unless the masters are damaged or a new design is required.

Sixteen hours: overnight silanization of microfabricated positive masters. critical step. do not proceed with sub-optimal silanization as the masters will be damaged

One hour: prepare negative masters using pdms. these negative masters can be used multiple times. if cared for properly they can be utilized over many months before this step will need to be repeated.

Sixteen hour: overnight silanization of negative pdms masters.

One hour: prepare IC and EC buffers for patching. CRITICAL STEP: ensure that buffers are precisely made and have the appropriate ph and osmolarity. poor buffers can lead to difficulties in forming a high resistance seal, opening the cell and maintaining a patch.

### Preparation Required for Each Experiment

Two hours: casting pillar arrays

One -sixteen hours: optional coating of arrays with ECM proteins

Thirty minutes: transfer of cells to pillar arrays

Thirty minutes -sixteen hours: culture of cells on arrays before use.

### Running the Experiment

Thirty minutes: preparation of patch pipettes, stimulating probe and initialization of patch-clamp rig for use, exchange of media for extracellular buffer on individual dish. CRITICAL STEP: poor pipettes will inhibit the ability to form a high resistance seal.

Two hours: maximum time any single sample should be analyzed

Five minutes: finding an appropriate cell, positioning the stimulating pipette, loading the patch pipette with IC buffer, positioning the patch pipette above the selected cell

Five seconds - Five minutes: obtaining a GΩ seal

Thirty seconds: compensating for fast and slow capacitive effects, checking patch parameters, setting a series resistance compensation

Thirty seconds: final positioning of stimulating probe

Two to five minutes: application of a series of stimuli (8–10) ranging from 1 nm−1 μm

Thirty minute: repositioning of stimulating probe adjacent a second appropriate pilus

Two to five minute: application of a series of stimuli (8–10) ranging from 1 nm−1 μm

Thirty minute: analysis of evoked currents and pillar deflections for each cell studied.

## Anticipated Results

Currents will be evoked by pillar movements in cells expressing MA channels that can be activated by substrate deflections and increasing stimuli result in currents of increasing amplitude (Figure 2). To date, deflection-activated currents have been evoked in cells expressing the mechanically activated channels PIEZO1, PIEZO2, and TRPV4 (Poole et al., 2014; Servin-Vences et al., 2017; Wetzel et al., 2017; Tay et al., 2018). In addition, currents were evoked by pillar deflection in primary sensory neurons and primary chondrocytes. The primary sensory neurons contained subsets of cells with differing mechanosensitivities: a population of low-threshold mechanoreceptors and a population of high-threshold nociceptors (Poole et al., 2014). Dedifferentiated chondrocytes exhibited a decrease in the activation threshold of deflection-evoked currents in comparison with primary chondrocytes themselves (Servin-Vences et al., 2017).

There are a number of ways to compare MA currents evoked using pillar arrays. Cells can be categorized as responsive vs. non-responsive to pillar deflections within the range 1–1,000 nm. Categorical data can be compared using Fisher's exact test. A minimum of 20 cells from each condition are required (Servin-Vences et al., 2017).

**Figure 4:**
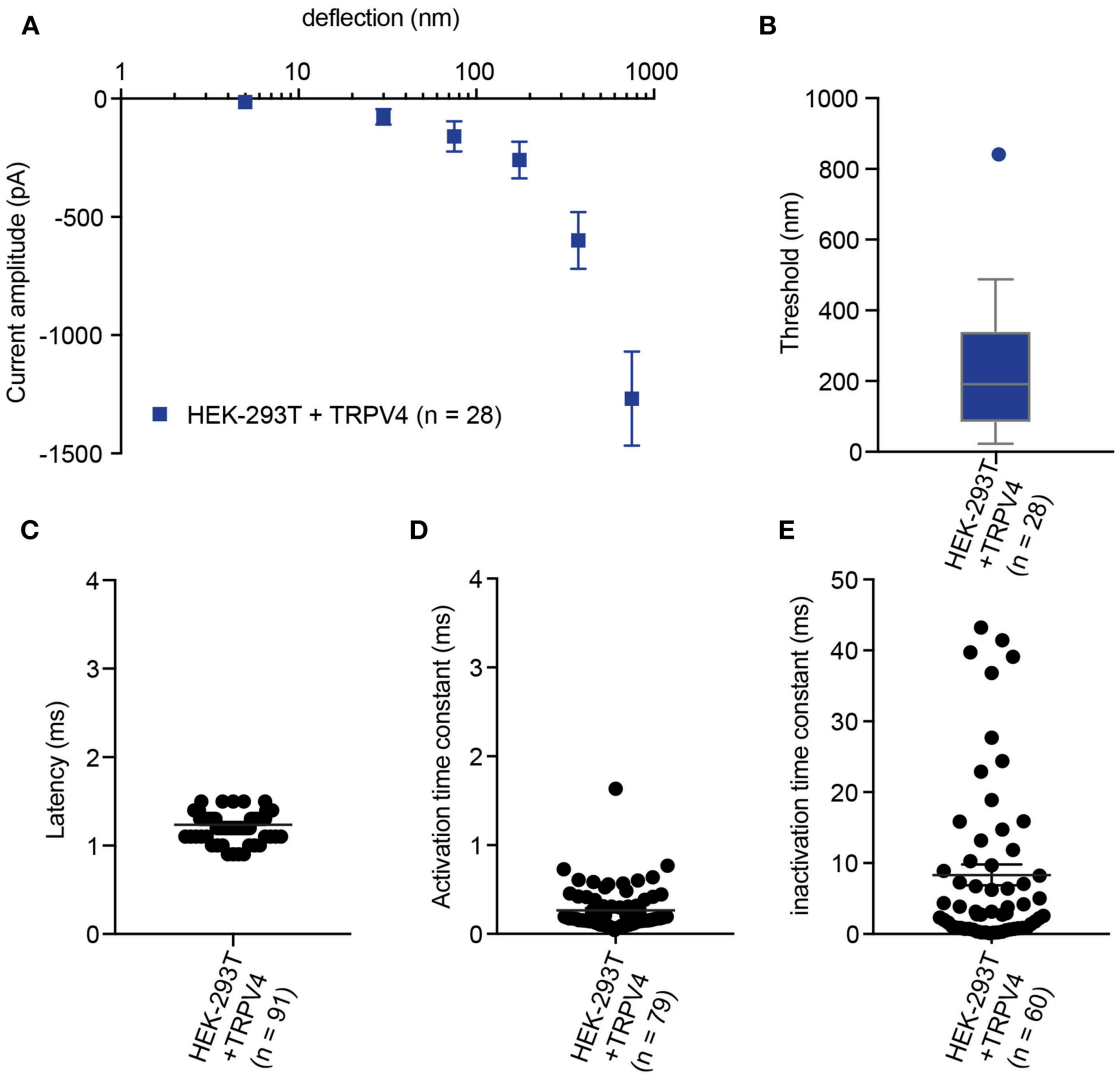
Representative data collected using HEK-293T cells expressing TRPV4**. (A)** Stimulus-response plot of TRPV4 currents induced by pillar deflections within the range 1–1,000 nm (*n* = 28 cells) **(B)** Activation threshold of TRPV4 to substrate deflection was calculated by averaging of smallest deflection that induced current in each cell (*n* = 28 cells). **(C)** Latency of deflection-activated TRPV4 currents. **(D)** Activation time constant of TRPV4 currents evoked by substrate deflection. **(E)** Inactivation time constant of TRPV4 currents evoked by substrate deflection.

**Figure 5:**
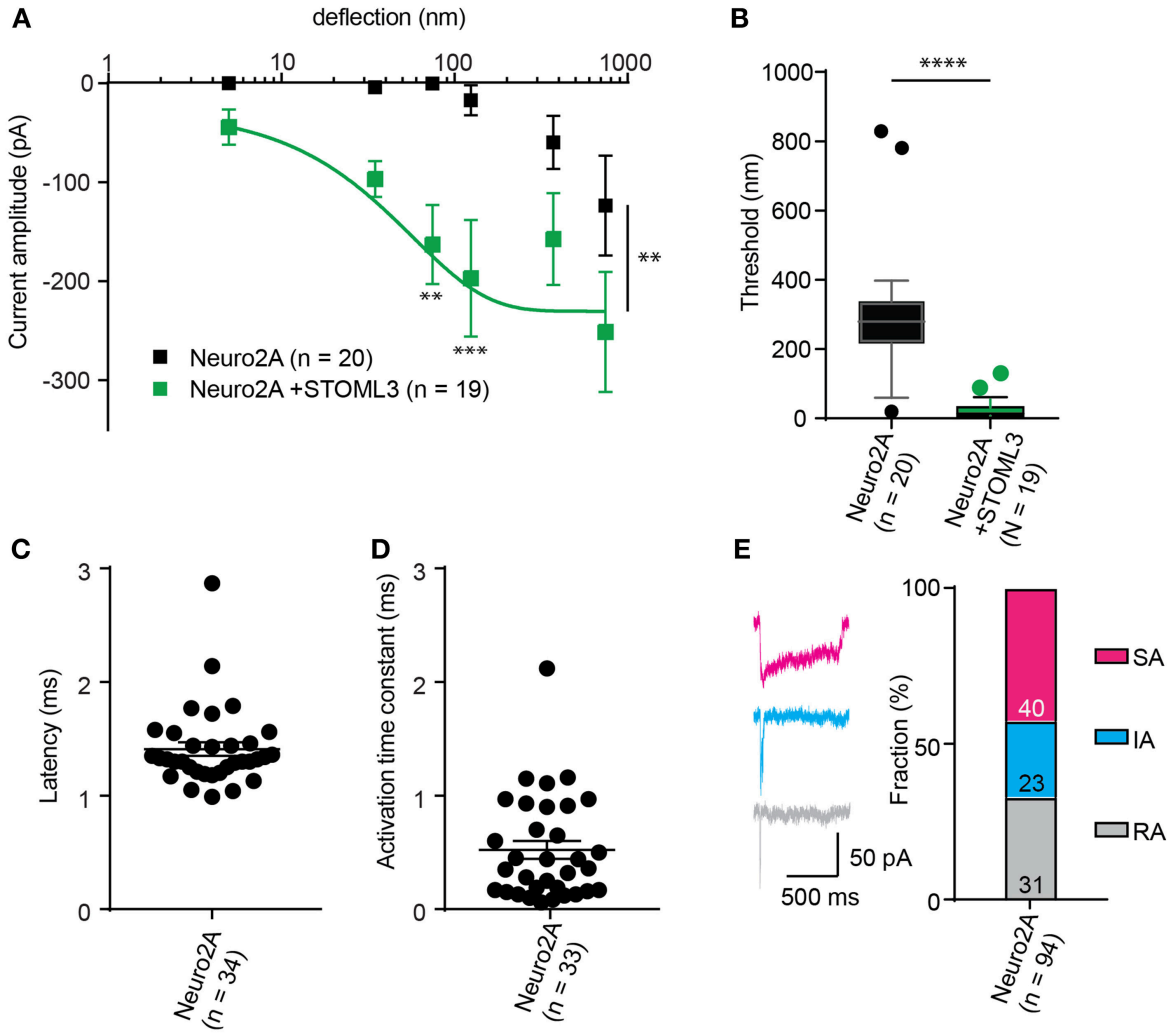
Representative data collected using Neuro2A cells. **(A)** Stimulus-response curve generated from Neuro2A cells (endogenously expressing PIEZO1). Overexpression of STOML3 in these cells leads to a sensitization of the MA currents (Ordinary Two-way ANOVA, *n* = 19 cells and 20 cells, respectively, curve comparison ^**^P = 0.0032, *post-hoc* analysis of individual bins ^**^P = 0.0043, ^***^P = 0.001). Boltzmann sigmoidal fit of Neuro2A + STOML3 data (green fit line) estimates a half maximal response of ~15 nm. **(B)** An analysis of the Neuro2A cells vs. Neuro2A cells + STOML3 indicates that STOML3 significantly reduces activation threshold of deflection-activated currents in these cells (Mann Whitney *U*-Test, ^****^P < 0.0001, *n* = 19 cells and 20 cells, respectively). Data plotted as box and whiskey plots, Tukey. **(C)** Latency of deflection activated currents in Neuro2A cells. **(D)** Activation time constant of deflection activated currents in Neuro2A cells. **(E)** Example traces of deflection activated currents in Neuro2A cells highlighting the variability in inactivation kinetics, classed as RA (τ_2_ < 5 ms), IA (5 < τ_2_ < 50 ms), or SA (τ_2_ > 50 ms or non-inactivating). Categorical plot of percentage of deflection activated currents in Neuro2A cells that are classed as RA, IA, or SA (numbers correspond to number of cells in each group, total *n* = 94). A subset of these data was previously published (Poole et al., 2014).

In order to calculate an activation threshold, average the smallest deflection that evokes a current for each cell. These data should be assessed to determine if they exhibit a normal distribution. Parametric data can be compared using a Student's *t*-test, non-parametric with a Mann-Whitney U test. Differences can be detected with approximately 15 cells per condition (Servin-Vences et al., 2017; Figures 4, 5).

The stimulus-response data collected from pillar array analysis have variation in x (deflection) and y (current amplitude); therefore, the response is grouped in bins of increasing stimulus size (0–10, 10–50, 100–250, 250–500, and 500–1,000 nm) in order to aid statistical comparison. For each cell, average the current amplitudes within each bin, and then average these data across cells. An ordinary two-way ANOVA with a Tukey *post-hoc* test can be used to statistically compare stimulus-response curves (Poole et al., 2014; Servin-Vences et al., 2017; Wetzel et al., 2017; Tay et al., 2018). We note that in most systems that we have tested, the stimulus-response curves of MA current amplitude exhibit large error bars. There are a number of likely reasons for this variability. The first is that the region to which stimuli are applied is delimited, corresponding to around 10 μm^2^, or less. As such the contact area between cell and substrate would only contain a restricted number of activatable channels which would result in noisier data. By using fluorimetric Ca^++^ imaging we can show that the initial influx of ions in response to pillar deflection occurs at the stimulated pilus, indicating that the channels that are activated are restricted to this region of the membrane (Figure S1). In addition, connections between the cell and the substrate will be dynamic, likely introducing confounding factors that influence the transfer of force from the deflected pilus to the channel, such as changes in cellular adhesion and localized cytoskeletal structures. The variation within the data thus likely reflects biological variability and if sufficient cells are analyzed, differences between groups can be determined.

The comparison of thresholds and stimulus-response curves can be used to test whether specific molecules or conditions affect the sensitivity of MA currents. For instance, we used pillar array analysis to demonstrate that the membrane scaffolding protein STOML3 sensitizes both PIEZO1 and PIEZO2. In addition, STOML3 is required for the molecular-scale sensitivity of touch receptive neurons (Poole et al., 2014). In some neuropathic pain states STOML3 levels are increased, presumably leading to hypersensitivity of these neurons and we showed that blocking STOML3 oligomerization can reverse neuropathic pain-driven behaviors in a number of mouse models (Wetzel et al., 2017). Here we have presented data that demonstrates the shift in sensitivity of MA currents in the presence of STOML3 and the reduction in transduction threshold (Figure 5).

The amplitude of currents evoked by pillar deflection in the most sensitive cells will saturate within the stimulus range, less sensitive cells do not. When the current amplitude does saturate the stimulus required for a half-maximal response can be determined from a Boltzmann sigmoidal fit of the data (calculated in GraphPad Prism 7.0) (Poole et al., 2014; Figure 5). In the case of touch receptive neurons or cells expressing PIEZO1 together with STOML3 the stimulus that results in a half maximal response is only ~15 nm (Poole et al., 2014; Figure 5). In fact, such a deflection is less than the width of a microtubule, representing an exquisite molecular-scale sensitivity to deflections of small regions of the membrane.

In addition to the sensitivity of the evoked currents, we also analyze the kinetic parameters of individual currents. Here were present kinetics for TRPV4 (Figure 4) and PIEZO1-mediated currents (Figure 5). The anticipated latency of channels directly activated by the mechanical input should be < 2 ms, and the activation time constant < 1 ms. Longer latencies indicate either inhibition of the force transfer to the channel or suggest a second messenger may be required for channel activation. Slower activation time constants suggest that the transfer of force to the set of activated channels is inhibited. We have observed that the inactivation time constants vary for distinct channels. Mechanically activated TRPV4 exhibits rapid inactivation kinetics (Servin-Vences et al., 2017; Tay et al., 2018; Figure 4), in contrast to current kinetics observed for the osmotic activation of TRPV4 (Lechner et al., 2011). For PIEZO1-mediated currents, we observe variable inactivation kinetics. We note that a significant fraction of PIEZO1-mediated currents are non-inactivating, in contrast to much of the data published for PIEZO1-mediated currents activated by HSPC and indentation. However, Gottlieb and colleagues noted that PIEZO1 channel inactivation kinetics were labile, as they also recorded non-inactivating currents (Gottlieb et al., 2012).

## Summary

The technical approach to evoking MA currents described here is a powerful tool to investigate MA channel activity within an intact cell-substrate interface. This approach preserves transmembrane structures, allowing an analysis of how MA channels function within a microenvironment that mimics cellular interfaces *in vivo*. In addition, the stimulus range of 1–1000 nm is consistent with the *in vivo* magnitude of deflections and movements that cells experience. This approach can also be used to study channels such as TRPV4 that are only mechanically activated by substrate deflections (but not indentation or membrane stretch) as well as to investigate how MA channels such PIEZO1 function within intact transmembrane structures. The quantitative nature of the experiments means that subpopulations of cells with variant mechanosensitivity can be identified and the molecules that tune MA channel activity can be analyzed. This technique is appropriate for the study of ion channel mediated mechanoelectrical transduction in those systems where mechanical inputs are propagated to the cell via the surrounding microenvironment. To date, this technique has been applied to primary somatosensory neurons (Poole et al., 2014; Wetzel et al., 2017) and primary chondrocytes (Servin-Vences et al., 2017). We propose that this approach would be also appropriate for the study of mechanoelectrical transduction in additional systems, such as in tumor cells and stem cells, where changes in the physical microenvironment can impact cellular function and the balance between physiological and pathophysiological states.

## Data Availability

All datasets generated for this study are included in the manuscript and the supplementary files.

## Author Contributions

Experiments were designed by KP and SS. Data were generated and analyzed by SS, AK, and EB. The manuscript was written by KP and SS with input from all of the authors.

### Conflict of Interest Statement

The authors declare that the research was conducted in the absence of any commercial or financial relationships that could be construed as a potential conflict of interest.
