# Supplementary material for: Analysis of Mechanically Activated Ion Channels at the Cell-Substrate Interface: Combining Pillar Arrays and Whole-Cell Patch-Clamp
Source: Front Bioeng Biotechnol. 2019 Mar 22;7:47. doi: 10.3389/fbioe.2019.00047 (PMC6448047; doi:10.3389/fbioe.2019.00047)
Supplement: Supplementary file 1 [file Table_1.DOCX]

| **Notes** | **Diameter (µm)** | **Height**  **(µm)** | **Center-to-center spacing (µm)** | **Image** |
| --- | --- | --- | --- | --- |
| 1, 2, 3 | 2 | 6 | 5 | 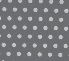 |
| 1, 3, 4 | 3.5 | 6 | 7 | 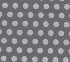 |
| 1 | 4.5 | 5 | 7.5 | 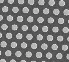 |
| 1 | 4.5 | 5 | 9 | 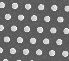 |
| 1 | 4.5 | 5 | 11.5 | 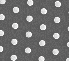 |
| 1 | 6.5 | 5 | 11.5 | 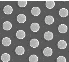 |

**Table S1: Validated dimensions of pillar arrays for evoking MA currents in adherent cells.**

Notes: 1, The values presented in this table represent the specifications of the design submitted for microfabrication of the positive master. The actual dimensions of the arrays should be analyzed using scanning electron microscopy (SEM) as the dimensions will differ from these specifications by up to approximately 10%. SEM. imaging will also indicate whether there are faults in the shape of the pili within the array. This SEM analysis need only be repeated once for each new array. 2, This specific array can be difficult to handle, the narrowness of the pillar elements and their packing result in the pili collapsing against each other. To minimise this effect the arrays can be removed from the master under 70% ethanol and should not be allowed to dry after this point. This approach means that the protocol (Section 3.4.3, *option 1*) for coating the tops of the arrays is no longer effective. 3, Data utilising this array published (Poole et al., 2014). 4, Data utilised using this array published (Servin-Vences et al., 2017; Wetzel et al., 2017). Scale bar = 10 μm.

Poole, K., Herget, R., Lapatsina, L., Ngo, H.-D., and Lewin, G. R. (2014). Tuning Piezo ion channels to detect molecular-scale movements relevant for fine touch. *Nat. Commun.* 5, 4520. doi:10.1038/ncomms4520.

Servin-Vences, M. R., Moroni, M., Lewin, G. R., and Poole, K. (2017). Direct measurement of TRPV4 and PIEZO1 activity reveals multiple mechanotransduction pathways in chondrocytes. *Elife* 6. doi:10.7554/eLife.21074.

Wetzel, C., Pifferi, S., Picci, C., Gök, C., Hoffmann, D., Bali, K. K., et al. (2017). Small-molecule inhibition of STOML3 oligomerization reverses pathological mechanical hypersensitivity. *Nat. Neurosci.* 20. doi:10.1038/nn.4454.
