# Supplementary figures and images for: Analysis of Mechanically Activated Ion Channels at the Cell-Substrate Interface: Combining Pillar Arrays and Whole-Cell Patch-Clamp
Source: Front Bioeng Biotechnol. 2019 Mar 22;7:47. doi: 10.3389/fbioe.2019.00047 (PMC6448047; doi:10.3389/fbioe.2019.00047)

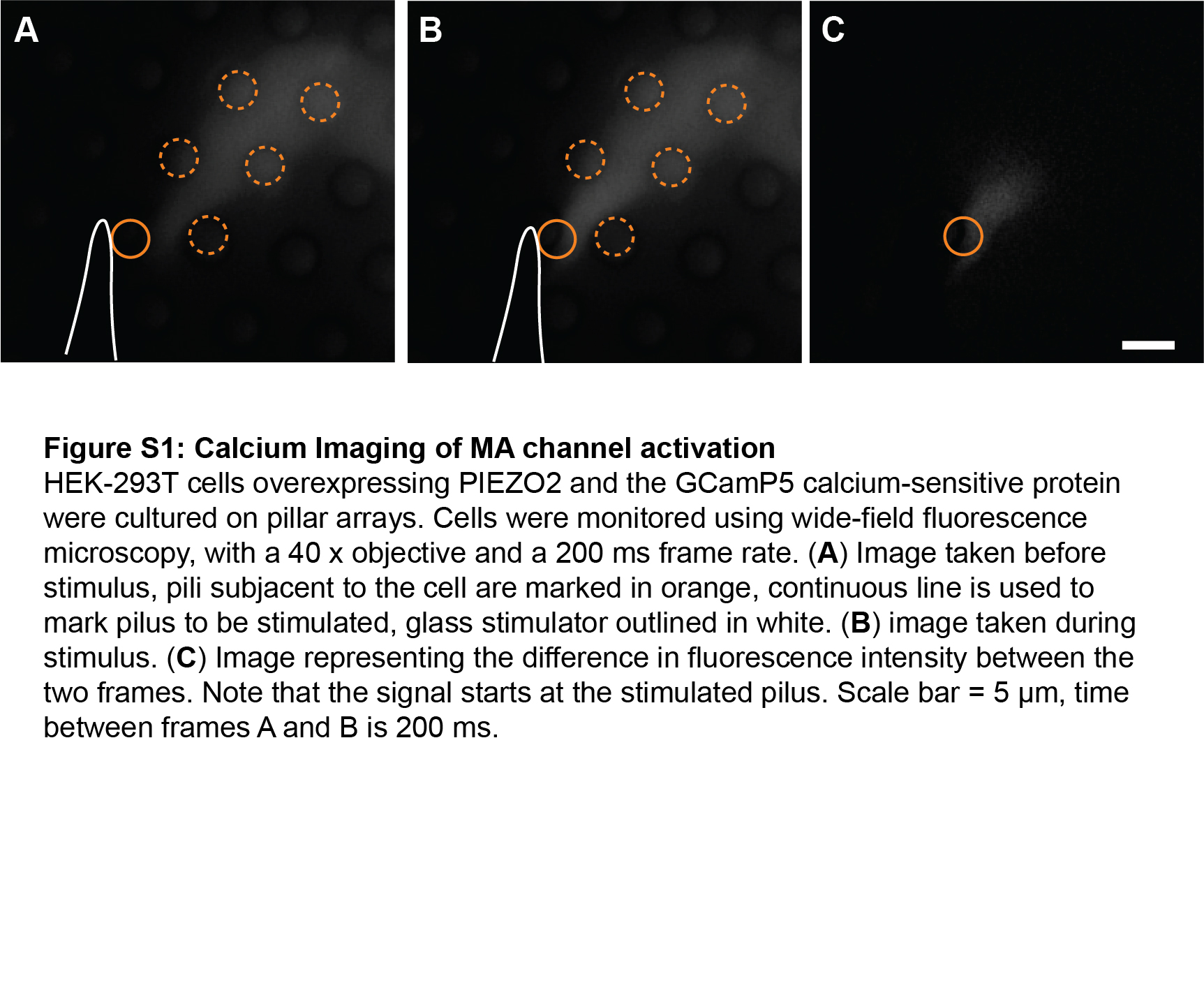

Supplement: Supplementary file 2 [file Image_1.JPEG]
